# Public–Private Partnerships as a Catalyst for Healthcare Transformation in Saudi Arabia: Evaluating the Impact on Accessibility, Quality, and Sustainability Under Vision 2030

**DOI:** 10.3390/healthcare14111435

**Published:** 2026-05-22

**Authors:** Salem Bauones, Mohammed J. Alsaadi

**Affiliations:** 1 Interventional Radiology Administration, King Fahad Medical City, Riyadh 11525, Saudi Arabia; sa.bauones@gmail.com; 2 Radiology and Medical Imaging Department, College of Applied Medical Sciences in Al-Kharj, Prince Sattam Bin Abdulaziz University, Al-Kharj 11942, Saudi Arabia

**Keywords:** public–private partnerships, Saudi Arabia, Vision 2030, healthcare accessibility, quality, sustainability

## Abstract

**Highlights:**

- The implementation of Public–Private Partnerships (PPPs) improved healthcare accessibility by expanding service coverage and integrating telemedicine.
- Financial barriers persisted, with many professionals reporting unchanged or increased out-of-pocket expenditures.
- Financial accessibility and reduced waiting times were significant predictors of patient satisfaction.
- Sustainability outcomes were moderately positive but constrained by workforce dependence, rural inequities, and reliance on subsidies.

**Implications of the study**

- Policy implication: Public–Private Partnerships (PPPs) can enhance healthcare accessibility under Vision 2030, particularly by expanding service capacity and integrating telemedicine.
- Health system implication: Persistent out-of-pocket costs indicate the need for stronger financial protection policies and regulatory oversight to ensure equitable access to PPP-delivered services.
- Sustainability implication: Long-term success of PPP healthcare models requires workforce nationalization, diversified financing strategies, and improved rural service distribution.

**Abstract:**

Background: PPPs are central to Saudi Arabia’s Vision 2030 healthcare transformation, yet evidence on their impact on accessibility, quality, and sustainability remains limited. The purpose of this study was to evaluate the perceived associations between PPP implementation under Vision 2030 and three healthcare system outcomes—service accessibility (geographical, financial, technological), care quality (clinical outcomes, patient satisfaction, efficiency), and reform sustainability (economic, operational, adaptive)—from the perspectives of healthcare professionals and patients in Saudi Arabia. Methods: A cross-sectional, mixed-methods design was employed. Surveys were administered to 150 healthcare professionals and 210 patients at PPP-operated facilities (response rates of 61.2% and 65.6%, respectively). Descriptive and inferential statistics—including *t*-tests, ANOVA, chi-square tests, and multiple regression analysis adjusted for age, sex, education, household income, comorbidities, and facility type were used to assess associations between PPP initiatives and outcomes. Instrument reliability was confirmed (Cronbach’s α ≥ 0.7), and content validity was supported by an expert-panel content validity index of 0.91. Thematic analysis of open-ended responses captured stakeholder perceptions and challenges (inter-coder κ = 0.83). Results: Among professionals, 56.6% reported improved accessibility following the implementation of PPP, with 60.6% endorsing telemedicine as a key facilitator. However, 64.6% indicated financial access remained unchanged or worsened due to persistent out-of-pocket expenditures, and a statistically significant urban–rural gap was observed (*p* = 0.008). Quality indicators showed positive trends, including improved patient outcomes (52%), reduced waiting times (60.6%), and high satisfaction with hygiene and safety (74%). Sustainability assessments were cautiously favorable (mean financial viability = 3.4/5), though subsidy dependence remained a concern. Adjusted regression analysis identified financial accessibility (β = 0.31, *p* < 0.001) and reduced waiting times (β = 0.23, *p* = 0.005) as variables significantly associated with patient-reported outcomes. Conclusions: PPPs were associated with measurable improvements in healthcare accessibility, quality, and efficiency in Saudi Arabia. However, achieving the Vision 2030 objectives requires reforms that address financial equity, service distribution, workforce nationalization, and governance.

## 1. Introduction

Healthcare systems worldwide face increasing pressures from demographic changes, a rising incidence of chronic illnesses, escalating technological costs, and growing demands for patient-centered care [1]. Saudi Arabia exemplifies these challenges: despite transforming from a limited to a comprehensive healthcare system over 40 years, with notable improvements in life expectancy and infant mortality [2], the Kingdom continues to grapple with rapid population growth, rising non-communicable disease burden, and ongoing urban–rural access disparities. Government expenditure exceeding 65% of total health spending further heightens sustainability concerns amid ongoing economic diversification away from oil dependence [3].

In response, Saudi Arabia’s Vision 2030 prioritizes healthcare reform, highlighting improvements in access, quality, safety, and financial sustainability [4]. Public–Private Partnerships (PPPs), defined as collaborative arrangements involving shared resources, risks, and responsibilities between the public and private sectors [5,6], have developed as a strategic tool within this reform plan. In healthcare, PPPs include private investment in infrastructure, facility management, and clinical service delivery under public oversight.

Global evidence on healthcare PPPs, however, shows mixed outcomes that are highly context dependent. In the United Kingdom, the Private Finance Initiative increased hospital capacity but later faced cost overruns [7]. In Lesotho, a flagship PPP hospital improved access to specialized care but consumed over half of the national health budget [8]. Similarly, PPPs in Turkey, Brazil, and India expanded infrastructure and rural access but raised concerns about affordability, equity, and long-term financial sustainability [9,10]. In Saudi Arabia, PPPs have enabled notable developments, including King Fahad Medical City and the expansion of primary care in underserved areas via telemedicine and electronic health records [11]. However, challenges remain, such as inequitable service distribution favouring urban populations, regulatory gaps that limit transparency and accountability, and heavy reliance on expatriate health workers, which threatens workforce sustainability [2,3,6].

Although PPPs are strategically central to Vision 2030, the international debate on healthcare PPPs has so far been dominated by case studies that report descriptive performance metrics without integrating equity, resource dependence, and performance assessment frameworks within a single analytical lens. This conceptual fragmentation is particularly consequential in oil-dependent emerging economies, where fiscal volatility, urban–rural inequities, and workforce nationalization pressures interact in ways that cross-national reviews seldom capture. The present study addresses this theoretical gap by combining the Health Equity Model, Resource Dependence Theory, and the OECD Health System Performance Assessment renewed framework within a mixed-methods empirical design applied to the Saudi healthcare reform under Vision 2030.

The purpose of this study, accordingly, is to evaluate the perceived associations between Public–Private Partnership (PPP) implementation under Vision 2030 and three healthcare system outcomes—accessibility, quality, and sustainability—from the perspectives of healthcare professionals and patients in Saudi Arabia, and to identify the factors most strongly associated with patient-reported outcomes and satisfaction within PPP-operated facilities. This study is guided by the following research question: To what extent have PPPs implemented under Vision 2030 improved healthcare accessibility, quality, and sustainability in Saudi Arabia, and what factors are most strongly associated with patient-reported outcomes and satisfaction? Four working hypotheses are tested: (H1) PPP implementation is associated with perceived improvements in healthcare accessibility, while persistent financial and geographic disparities remain; (H2) PPP implementation is associated with perceived improvements in care quality, particularly through technological adoption and reduced waiting times; (H3) PPP-driven reforms are perceived as cautiously sustainable, with subsidy dependence and workforce challenges acting as moderators; and (H4) financial accessibility and reduced waiting times are the strongest correlates of patient-reported outcomes and satisfaction within PPP-operated facilities.

The study contributes to the international debate by (i) producing one of the first empirical, mixed-methods evaluations of healthcare PPPs in the Gulf Cooperation Council region under a comprehensive national reform programme; (ii) suggesting which PPP modalities (infrastructure investment, facility management, and clinical service delivery under public oversight) appear more effective in oil-dependent emerging economies; and (iii) identifying a parsimonious set of policy-evaluation indicators that may facilitate comparative analyses across heterogeneous healthcare systems.

## 2. Literature Review and Theoretical Framework

Healthcare PPPs encompass a heterogeneous set of contractual arrangements through which public authorities and private actors share resources, risks, and responsibilities to deliver health-related infrastructure or services. The international literature distinguishes three main modalities: (i) infrastructure-based PPPs, in which the private partner finances, designs, builds, and maintains health facilities; (ii) facility-management PPPs, focused on non-clinical services such as logistics, catering, and information systems; and (iii) integrated clinical-service PPPs, in which the private partner also delivers clinical care under public oversight [5,6,10]. Each modality entails distinct trade-offs in equity, efficiency, and accountability.

Empirical evidence on healthcare PPPs is mixed. Ferreira and Marques reported that Portuguese PPP hospitals achieved shorter waiting times and lower error rates than comparable public facilities, while raising concerns about long-term contractual flexibility [12]. Casprini and Palumbo documented that digital PPPs in Italy expanded patient reach by 15% but required strong governance to translate technological adoption into sustained quality gains [13]. Joudyian et al., in a scoping review of PPPs in primary healthcare, concluded that effects on quality and access are highly context-dependent and frequently constrained by weak monitoring systems [14]. Evaluations of Lesotho’s national referral hospital PPP highlighted improved technical efficiency alongside fiscal pressures that ultimately proved unsustainable [15,16]. Studies from India, Pakistan, and Bangladesh have shown that PPP-supported telemedicine and mobile-clinic initiatives can extend services to rural areas, but that sustainability depends on continuous public funding and clear performance benchmarks [17,18].

In the Gulf region, the literature remains comparatively thin. Alasiri and Mohammed [19] and AlHanawi and Qattan [11] have examined the institutional environment of Saudi PPPs, while Suleiman and Ming [20] reviewed the Vision 2030 healthcare model. However, these contributions are predominantly conceptual and rarely combine quantitative and qualitative evidence from healthcare professionals and patients. The present study, therefore, fills a clear empirical and theoretical gap.

**Figure 1:**
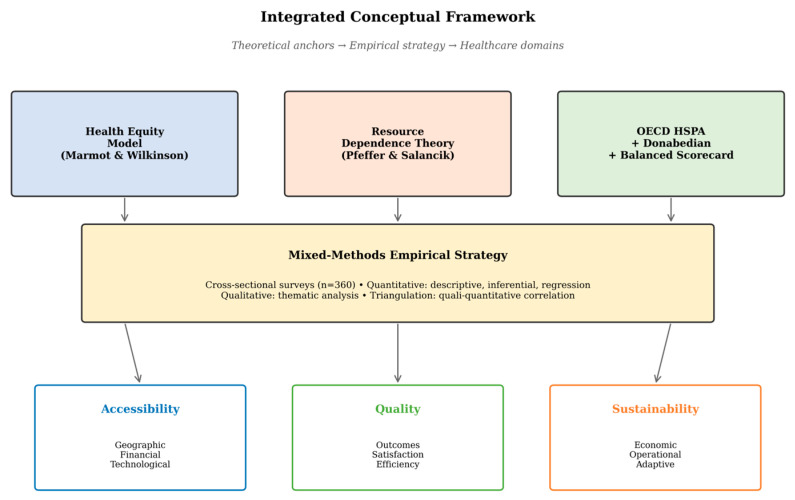
Integrated conceptual framework linking the Health Equity Model, Resource Dependence Theory, and the OECD Performance Assessment framework with the three healthcare domains evaluated (accessibility, quality, sustainability).

Three theoretical anchors guide the present analysis. First, the Health Equity Model [21] frames accessibility as multidimensional, comprising geographic, financial, technological, and informational components, each shaped by socioeconomic determinants. Second, Resource Dependence Theory [22] interprets PPPs as inter-organsational arrangements through which public actors mitigate fiscal vulnerability by mobilising private capital, technology, and managerial capacity. Third, the OECD Health System Performance Assessment renewed framework [23,24] complemented by the Donabedian structure–process–outcome model and the Kaplan–Norton balanced scorecard [25] provides the conceptual basis for evaluating quality and sustainability across structural, process, and outcome dimensions. The integrated conceptual model that aligns these three anchors with the empirical strategy of the present study is depicted in Figure 1.

## 3. Materials and Methods

### 3.1. Study Design

This study used a cross-sectional, mixed-methods design, combining quantitative and qualitative assessments to evaluate the impact of PPPs in healthcare on accessibility, quality, and sustainability in Saudi Arabia. The quantitative component involved structured survey data from healthcare professionals and patients, while the qualitative component comprised open-ended survey responses analysed thematically. Reporting follows the STROBE guidelines for cross-sectional studies (the completed STROBE checklist is provided as Supplementary Materials).

Data were collected through structured online surveys distributed to healthcare professionals and patients at facilities operating under PPP models. All quantitative analyses were conducted in IBM SPSS Statistics version 29; thematic coding was managed in NVivo 14; and visualisations were produced in R version 4.4 (ggplot2 package).

### 3.2. Participants, Sampling, and Recruitment

A stratified-pragmatic (quota-based) sampling design with convenience elements was used. Pre-defined quotas were set across the five administrative regions of Saudi Arabia (Central, Western, Eastern, Northern, and Southern) and across facility types (urban/rural, government/private, primary/secondary/tertiary). Still, recruitment within each stratum proceeded through institutional gatekeepers and online channels. Although this approach approximates a stratified design, it does not constitute true probabilistic random sampling, and the corresponding limitations are explicitly acknowledged in Section 5.5.

The sampling frame comprised (a) registered healthcare professionals (doctors, nurses, hospital managers, technicians, pharmacists, and policymakers) at participating PPP-operated facilities and (b) adult patients (≥18 years) who had received care in such facilities within the previous 12 months. Inclusion criteria were active employment in or use of a PPP facility; ability to read Arabic or English; and provision of informed consent. Exclusion criteria were incomplete responses, duplicate IP addresses, and respondents not affiliated with PPP facilities.

**Figure 2:**
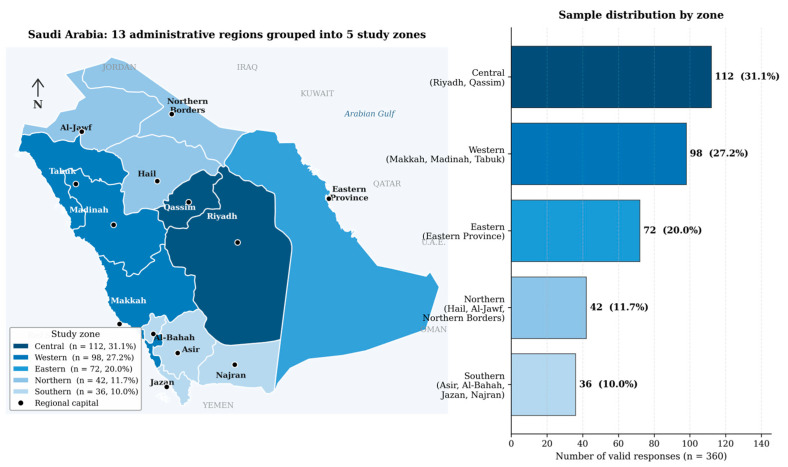
Geographic dispersion of valid questionnaires across Saudi Arabia (n = 360). The 13 official administrative regions are colour-coded according to the five study zones used for stratification. Source: Original figure created by the authors from primary survey data (2026); base map outlines derived from the open-source @svg-maps/saudi-arabia dataset (MapSVG, CC-BY-4.0).

**Figure 3:**
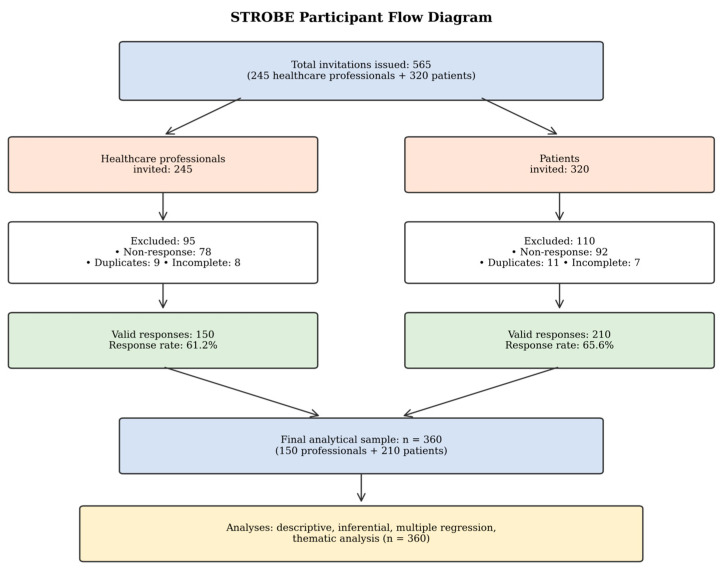
Participant flow diagram (STROBE format) showing recruitment, exclusions, and final analytical samples.

In total, 245 questionnaires were distributed to healthcare professionals (150 valid responses; response rate 61.2%) and 320 to patients (210 valid responses; response rate 65.6%). Within each stratum, invitations were issued in random order from gatekeeper-supplied registers, and survey dissemination was time-staggered to minimize selection bias. Geographic dispersion of valid responses across the five administrative regions is shown in Figure 2, and a STROBE-style participant-flow diagram is presented in Figure 3. It should be noted that the term “healthcare professionals” is used inclusively in this study and encompasses not only clinical staff but also hospital managers, government officials, policymakers, and researchers affiliated with PPP-operated facilities.

### 3.3. Sample Size and Power Analysis

An a priori power analysis was conducted using G*Power 3.1. With *α* = 0.05 and power (*1* − *β*) = 0.80, the minimum sample sizes required to detect medium effects were 128 for an independent-samples *t*-test (Cohen’s d = 0.5), 159 for a one-way ANOVA across three groups (Cohen’s *f* = 0.25), and 103 for a multiple regression model with up to six predictors (Cohen’s *f^2^* = 0.15). The achieved samples of 150 healthcare professionals and 210 patients, therefore, provide adequate statistical power for all planned analyses. A post hoc sensitivity analysis indicated that the achieved patient sample (n = 210) is sensitive to detect a regression effect of *f^2^* ≥ 0.07 with 80% power, supporting the adequacy of the regression analyses.

### 3.4. Survey Content and Instrument Development

The survey was divided into five sections, with different questions for healthcare professionals and patients: Participant Demographics; Accessibility of Healthcare Services; Quality of Healthcare Services; Sustainability of PPP-Driven Healthcare Services; and Open Feedback and Recommendations. The survey combined Likert-type, multiple-choice, and open-ended questions to capture both quantitative and qualitative data.

Items were adapted from validated instruments used in prior PPP and health-services research [12,13,14] and refined for the Saudi context. Content validity was assessed by a panel of seven experts (three health-policy researchers, two senior PPP managers, and two practising clinicians); the average content validity index (CVI) was 0.91. The questionnaire was forward- and back-translated between English and Arabic by two independent bilingual researchers, with discrepancies resolved by consensus. A pilot test was conducted with 28 respondents (15 professionals, 13 patients) not included in the final sample; their feedback informed wording refinements and the removal of two ambiguous items. Construct validity was further supported by exploratory factor analysis on the pilot data, in which the three-factor structure (accessibility, quality, sustainability) accounted for 64.8% of the variance. The full questionnaire is provided in Appendix A.

### 3.5. Data Analysis

#### 3.5.1. Quantitative Analysis

For each predictor, both the unstandardised regression coefficient (B) and the standardised coefficient (β) are reported. B indicates the change in the patient-reported composite outcome score (measured on the original 1–5 Likert scale) associated with a one-unit change in the predictor, holding all other variables constant. β expresses the same association after all variables have been rescaled to unit variance and is therefore directly comparable across predictors measured on different scales. The 95% confidence interval, t-value, *p*-value, and Variance Inflation Factor (VIF) are also reported.

Descriptive statistics (frequencies, percentages, means, and standard deviations) summarised perceptions of accessibility, quality, and sustainability. Comparative statistical analyses were conducted using *t*-tests and ANOVA to assess variation across regions, demographics, and healthcare settings. Chi-square tests were used to examine associations between categorical variables. Multiple regression analysis was performed to identify variables associated with perceived changes in healthcare outcomes under PPPs. Internal consistency of the survey instruments was evaluated using Cronbach’s alpha, with α ≥ 0.70 considered acceptable.

Predictors for the regression models were selected based on (a) theoretical relevance derived from the literature reviewed in Section 2 and (b) statistical significance in bivariate screening at *p* < 0.10.

The four core predictors entered into the multivariable regression model (financial accessibility, ease of access, reduced waiting times, and distance from facility) correspond directly to the equity and access dimensions of the integrated theoretical framework presented in Section 2 (Figure 1). Specifically, financial accessibility and distance map onto the financial and geographic dimensions of the Health Equity Model; reduced waiting times and ease of access correspond to the process and access dimensions of the OECD Health System Performance Assessment framework; and the inclusion of facility type as a covariate operationalises the inter-organisational logic of Resource Dependence Theory. Path analysis and structural equation modelling are acknowledged as appropriate next steps and are listed among the priorities for further research in Section 6.

The multiple regression models adjust for age, sex, education level, monthly household income (as a proxy for socioeconomic status), self-reported comorbidity count, and facility type (government vs. private). Variance Inflation Factors (VIF) were inspected to detect multicollinearity (acceptable threshold VIF < 5).

To exploit the heterogeneity of the sample, three pre-specified subgroup dimensions were considered: (i) geographic location (urban vs. rural; distance band <10 km, 10–30 km, >30 km); (ii) facility type (government vs. private; primary, secondary, tertiary); and (iii) utilisation frequency (rare, occasional, frequent). These dimensions, motivated by the Health Equity Model and Resource Dependence Theory, informed the inferential and stratified regression analyses presented in Section 4.

Missing-data handling: pairwise deletion was used for descriptive analyses; multiple imputation by chained equations (MICE; m = 20) was applied to the regression models. Sensitivity analyses comparing the imputed-data results with complete-case analyses produced consistent point estimates.

#### 3.5.2. Qualitative Analysis

Open-ended responses were analyzed using thematic analysis, involving coding, theme development, and interpretation, to identify common barriers to PPP implementation and to generate stakeholder recommendations for improvement.

Specifically, two researchers independently coded all open-ended responses using a six-phase thematic analysis procedure [24] conducted in NVivo 14. Inter-coder reliability was assessed using Cohen’s kappa, yielding κ = 0.83 (95% CI 0.78–0.87), indicating strong agreement. Discrepancies were resolved through discussion and, where necessary, with the involvement of a third researcher. A reflexivity statement is included in the Supplementary Materials. The qualitative component of this study is reported in adherence to the Standards for Reporting Qualitative Research (SRQR) [26] and the Consolidated Criteria for Reporting Qualitative Research (COREQ) [27]. A completed SRQR checklist is provided in the Supplementary Materials; relevant COREQ items (research team and reflexivity, study design, and analysis and findings) are addressed throughout Section 3.5 and Section 4.7.

### 3.6. Quali-Quantitative Triangulation

Convergent validity between the qualitative and quantitative strands was assessed by computing the Spearman rank correlation between the relative frequency of qualitative themes and the corresponding mean Likert scores of the related quantitative dimensions. This rank correlation indicates the extent to which thematic salience in the qualitative responses aligns with the order of mean dimension scores in the quantitative analysis.

### 3.7. Ethical Considerations

The study was conducted in accordance with the Declaration of Helsinki and approved by the Institutional Review Board of King Fahad Medical City (IRB Log Number 25-7001, dated 16 February 2026). Informed electronic consent was obtained from all participants before the survey commenced. All responses were anonymous, and no identifying information was collected. Data were stored on a password-protected institutionally controlled server accessible only to the research team and will be retained for five years in compliance with the Saudi Personal Data Protection Law (PDPL). Participants could withdraw at any time without consequence.

## 4. Results

### 4.1. Participant Demographics

A total of 360 respondents participated in this study, including 150 healthcare professionals and 210 patients recruited from PPP-operated healthcare facilities across Saudi Arabia. Among the professional group, most were healthcare practitioners (60.7%, n = 91), followed by policymakers (8.7%, n = 13), government officials (8.7%, n = 13), researchers (6.0%, n = 9), hospital managers (4.0%, n = 6), nurses (2.0%, n = 3), and technicians (0.7%, n = 1). The mean professional experience level was 3.7 on a 6-point ordinal scale (SD = 1.6), equivalent to roughly 10–11 years, indicating a balanced representation across early-, mid-, and late-career stages. Most professionals worked in the government sector (65.3%, n = 98), with the remaining in the private sector (34.7%, n = 52), reflecting the central role of public healthcare within PPP frameworks in Saudi Arabia.

Among the patient cohort, the frequency of healthcare utilization was distributed as follows: 33.3% (n = 70) accessed services often, 26.7% (n = 56) very frequently, 23.3% (n = 49) sometimes, and 16.7% (n = 35) rarely (M = 2.7, SD = 1.1). With 60.0% of patients classified as frequent users, the findings are particularly relevant for chronic-care and regular-utilization contexts, while still capturing the perspectives of occasional users.

### 4.2. Instrument Reliability

The internal consistency of the survey instruments was assessed using Cronbach’s alpha (α). All subscales across both cohorts met or exceeded the widely accepted threshold of α ≥ 0.70. Table 1 presents the item-level statistics—corrected item–total correlations, Cronbach’s alpha if the item is deleted, item means, and standard deviations—for each of the three domains (accessibility, quality, sustainability), as well as the overall subscale alpha for each cohort. The quality subscale demonstrated the highest reliability in both groups (α = 0.82), while sustainability showed the lowest yet acceptable values (professionals: α = 0.71; patients: α = 0.74). 

### 4.3. Accessibility

Among healthcare professionals, 56.6% reported improved ease of access after PPP implementation, with 17.3% (n = 26) indicating access became “much easier” and 39.3% (n = 59) “somewhat easier” (M = 3.2, SD = 1.1). Telemedicine was regarded as beneficial by 60.6% of respondents (M = 2.7, SD = 0.8). However, financial accessibility remained a significant gap: 43.3% reported no change in out-of-pocket costs, 21.3% experienced increased costs, and only 35.3% reported a reduction in financial burden (M = 2.3, SD = 1.0).

**Figure 4:**
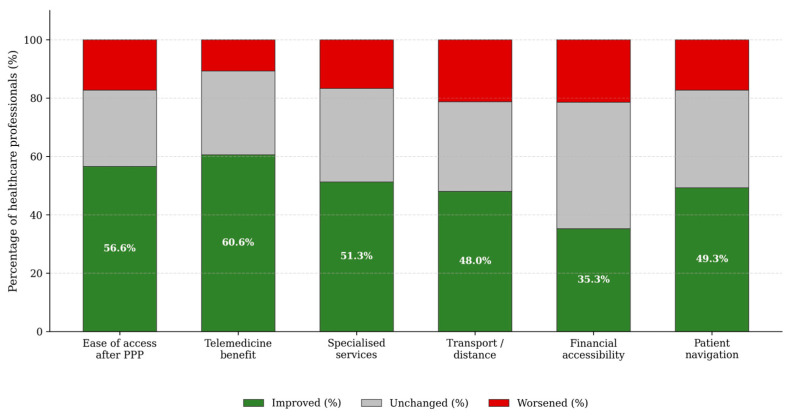
Healthcare professionals’ perceptions of accessibility indicators following PPP implementation (n = 150).

Patient responses showed a similar pattern, with 53.4% reporting improved access (M = 2.5, SD = 1.2) and 60.0% endorsing telemedicine (M = 2.7, SD = 0.9). Financial affordability was equally concerning among patients, with 63.3% reporting unchanged or increased costs (M = 2.2, SD = 1.0). The lower patient mean for ease of access (2.5 vs. 3.2) and higher variability (SD = 1.2) reflect geographical disparities in access—a finding supported by the inferential analysis showing a statistically significant urban–rural difference (M = 2.8 vs. 2.1; *p* = 0.008). These results suggest that, while PPPs were associated with perceived gains in physical and technological access, financial barriers and rural inequities have not been resolved and remain visible across the dataset (Figure 4).

### 4.4. Quality of Care

**Figure 5:**
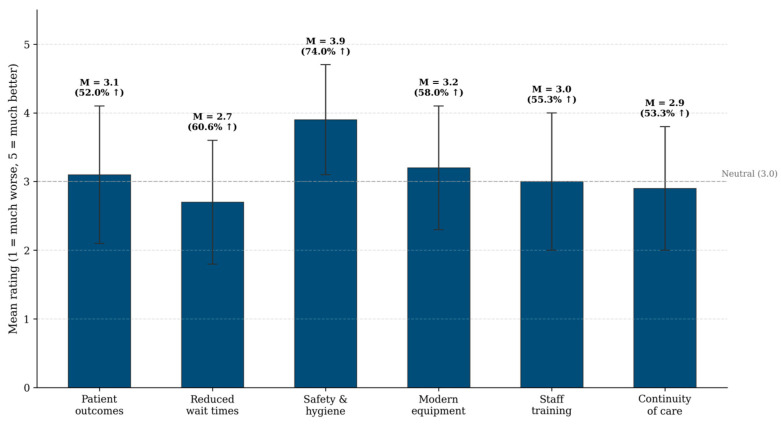
Healthcare professionals’ perceptions of quality indicators in PPP facilities (n = 150).

Quality indicators showed consistently positive trends. Among healthcare professionals, 52.0% reported improved patient outcomes (M = 3.1, SD = 1.0), 60.6% reported shorter wait times (M = 2.7, SD = 0.9), and 74.0% expressed satisfaction with safety and hygiene standards (M = 3.9, SD = 0.8)—the highest-rated indicator in the entire study (Figure 5). The low variability in safety satisfaction (SD = 0.8) indicates strong, uniform adherence to safety protocols across PPP facilities. Patient-reported quality was similarly favourable: 60.0% noted improved outcomes (M = 2.7, SD = 1.0), 66.6% experienced reduced wait times (M = 2.8, SD = 0.9)—the strongest patient-reported quality indicator—and 63.3% were satisfied with safety standards (M = 2.8, SD = 1.0). Notably, patients reported higher rates of improvement for both outcomes (60.0% vs. 52.0%) and wait times (66.6% vs. 60.6%) than professionals did, likely reflecting the direct experiential benefits of service delivery improvements.

### 4.5. Sustainability

**Figure 6:**
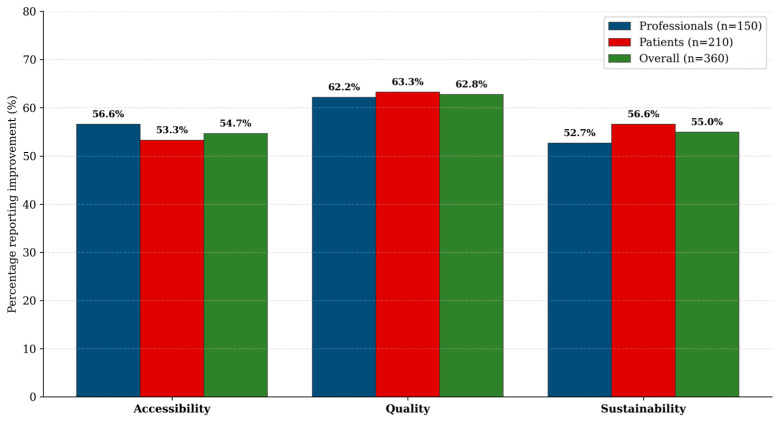
Comparative perceptions of PPPs across healthcare domains: professionals vs. patients.

Perceptions of sustainability were cautiously optimistic across both groups. Financial viability was rated positively by 52.7% of professionals (M = 3.4, SD = 1.0) and 56.6% of patients (M = 2.7, SD = 1.0), although around 44% of professionals remained neutral or expressed concerns about long-term funding sustainability. Operational efficiency was seen as improved by 56.6 % of professionals (M = 2.7, SD = 0.9) and 53.3% of patients (M = 2.6, SD = 1.0). Resilience and adaptability received the lowest ratings from both groups (professionals: M = 2.6; patients: M = 2.7), with about 40% expressing neutral or negative views, indicating limited confidence in PPPs’ crisis response and long-term adaptive capacity. When combined as a sample-size-weighted average across the two cohorts (n = 360), the overall reported improvements were 54.7% for accessibility, 62.8% for quality, and 55.0% for sustainability, with quality standing out as the strongest area of perceived PPP impact (Figure 6).

### 4.6. Inferential and Regression Analyses

Inferential analyses on the patient dataset (n = 210) revealed statistically significant differences across demographic and utilization subgroups (Table 2). Independent samples *t*-tests confirmed that urban patients reported significantly greater ease of access than rural patients (M = 2.8 vs. 2.1; *p* = 0.008), and frequent healthcare users reported higher safety satisfaction than infrequent users (M = 2.9 vs. 2.6; *p* = 0.038). One-way ANOVA showed that perceptions of financial viability decreased progressively with increasing distance from facilities (F = 4.35, *p* = 0.005). Patient-reported outcomes also differed significantly by utilization frequency (F = 3.25, *p* = 0.023), with rare users reporting the lowest perceived improvement (M = 2.5). Chi-square tests revealed significant associations between safety satisfaction and distance (χ^2^ = 6.15, *p* = 0.013), with 80% of urban patients reporting satisfaction compared to 50% of rural patients, and between telemedicine impact and healthcare frequency (χ^2^ = 7.02, *p* = 0.008), with frequent users showing higher positive perceptions of telemedicine (55.6%) than infrequent users (45.0%).

Financial affordability emerged as the strongest variable associated with patient-reported outcomes (β = 0.31, *p* < 0.001), indicating a positive standardized association between perceived financial accessibility and patient-reported outcomes within the model (Table 3). Ease of access (β = 0.27, *p* = 0.002) and reduced waiting times (β = 0.23, *p* = 0.005) were also significantly associated with the outcome. Distance had a significant negative association (β = –0.16, *p* = 0.024), confirming that geographical remoteness is associated with lower patient-perceived outcomes. A supplementary regression model on satisfaction identified ease of access as the variable most strongly associated with satisfaction (β = 0.48, *p* < 0.001), followed by financial accessibility (β = 0.31, *p* = 0.002). 

Predictors selected based on theoretical relevance and bivariate screening (*p* < 0.10). VIF < 5 indicates the absence of problematic multicollinearity. Stratified regression models were estimated for the urban vs. rural and government vs. private subsamples to explore heterogeneity in the strength of the predictors. Table 4 reports the standardized coefficients of the four core predictors across the four subsamples, illustrating that financial affordability was the dominant correlate among rural and private-facility patients, whereas reduced waiting times had a stronger relative weight among urban and government-facility patients.

### 4.7. Qualitative Thematic Analysis

**Figure 7:**
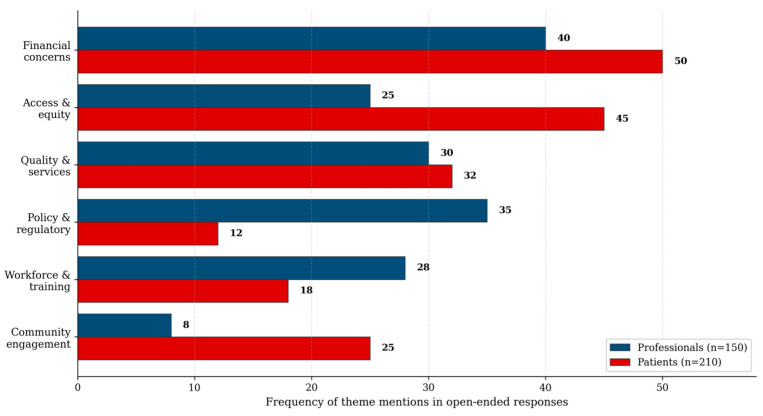
Qualitative thematic analysis: frequency of key themes by stakeholder group.

Thematic analysis of open-ended survey responses identified six main themes across both stakeholder groups (Figure 7). Financial concerns were the most frequently mentioned theme by both professionals (~40 mentions) and patients (~50 mentions), with patients emphasizing immediate costs such as health insurance and transparency, while professionals focused on systemic sustainability issues, including ongoing funding and project delays. This overlap aligns with the quantitative results, in which financial accessibility had the lowest mean across both groups (M = 2.2–2.3). Access and equity were discussed considerably more by patients (~45 mentions) than professionals (~25 mentions), aligning with the statistically significant urban–rural access gap (*p* = 0.008). Policy and regulatory issues were raised primarily by professionals (~35 mentions), reflecting their focus on systemic governance, while community engagement and health education were prioritized mainly by patients (~25 mentions).

Representative anonymized excerpts illustrate the principal themes. On financial concerns, a patient observed: “Even with PPP services, the out-of-pocket costs remain a barrier for my family.” On access and equity, a rural respondent stated: “The new clinic is much closer than before, but specialist care still requires travelling to the city.” On telemedicine, a professional remarked: “Virtual consultations have reduced the load on outpatient clinics, but the rural broadband infrastructure is uneven.” On governance and regulation, a hospital manager noted: “Performance metrics for the partnership are not always clear, which complicates accountability.” On community engagement, a patient commented: “More awareness sessions would help patients understand what PPP services include and what they cost.” On workforce and training, a professional explained: “Continuing education opportunities have improved, yet workload pressures limit attendance.”

Regarding perceived alignment with Vision 2030, approximately 50% of patients characterized PPP initiatives as “very aligned” with national healthcare objectives, compared with only 15% of professionals; most professionals (60%) rated alignment as “somewhat aligned.” This divergence suggests that professionals’ awareness of implementation challenges, including project delays and regulatory ambiguity, tempers their optimism compared with patients’ experiences of tangible service improvements. The quali-quantitative correlation analysis—operationalized as the Spearman rank correlation between thematic frequencies and the corresponding mean Likert scores of the related quantitative dimensions—yielded moderate convergence (r = 0.58) and a strong rank correlation (ρ = 0.80), supporting the convergent validity of the integrated findings.

## 5. Discussion

**Figure 8:**
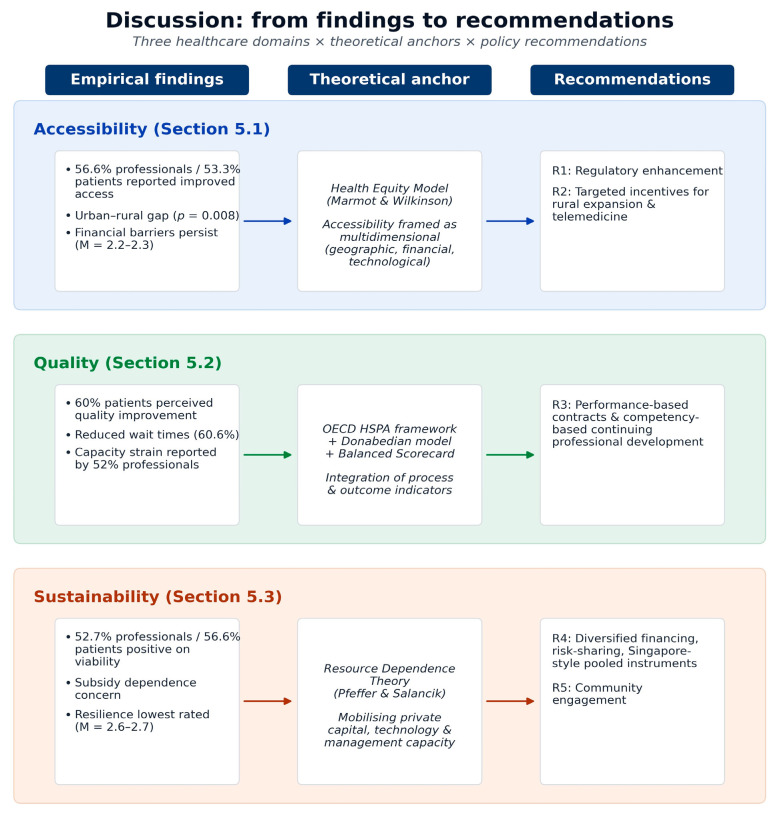
Conceptual scheme of the discussion: linkage between empirical findings, theoretical anchors, and recommendations across the three healthcare domains. Source: Authors’ elaboration.

This study explored the role of Public–Private Partnerships (PPPs) in reshaping Saudi Arabia’s healthcare system under Vision 2030, with a focus on accessibility, quality, and sustainability. The findings indicate that PPPs were associated with measurable perceived improvements across these areas, although significant challenges remain that warrant careful attention. This discussion places the results within the broader global evidence base, highlights key gaps, and provides evidence-based recommendations for policymakers and researchers aiming to optimise PPP implementation in the Saudi healthcare context. Figure 8 provides a conceptual scheme mapping each of the three discussion subsections (accessibility, quality, sustainability) to their corresponding empirical findings, theoretical anchors, and recommendations, offering a visual roadmap for the argument that follows.

### 5.1. Healthcare Accessibility and Public–Private Partnerships

The finding that 56.7% of professionals and 53.3% of patients reported improved healthcare accessibility following the implementation of PPPs represents a substantial step towards Vision 2030′s universal-coverage goal [28]. Notably, however, this perceived gain coexists with persistent financial barriers (mean financial accessibility = 2.2–2.3) and a statistically significant urban–rural gap (*p* = 0.008), meaning that the accessibility narrative cannot be read in isolation from these inequities. This pattern aligns with global evidence showing that PPPs extend coverage to geographically isolated and marginalised populations [29]. In India, PPP-supported mobile health units and telemedicine platforms increased patient coverage by 10–40% while reducing travel burdens [17,18]. Similarly, Portugal’s PPPs resulted in a 15% reduction in wait times (*p* < 0.05) [16], and scoping reviews of primary-healthcare PPPs worldwide report a 35% increase in the reach of preventive services [30]. In Saudi Arabia, PPP-funded primary healthcare centres represent a deliberate shift from an urban-centric, centralised model to a more inclusive framework that uses private-sector flexibility under public oversight to reduce persistent urban–rural disparities [20].

Accessibility improvements remain uneven, however, especially in rural regions where infrastructure gaps—poor road networks, limited broadband access, and logistical challenges—continue to hinder service provision. This disparity is well recognised globally: in sub-Saharan Africa, PPP-driven access depends on local governance capacity and ongoing funding [31], whereas South Africa’s urban PPP hospitals perform well, but rural programmes struggle due to unreliable infrastructure [32]. The Health Equity Model [21] highlights that fair access requires addressing socioeconomic factors—education, income, and transportation—beyond physical infrastructure alone. In Saudi Arabia, PPPs have yet to fully resolve these structural issues. Digital PPPs offer a partial remedy; Italy’s Lab@AOR project increased patient reach by 15% through telehealth [13], suggesting that Saudi Arabia could implement solar-powered mobile clinics, expand broadband coverage, and involve community health workers to improve last-mile delivery [19], so that access is not confined to urban, wealthier populations.

### 5.2. Impact of PPPs on Healthcare Quality

Quality indicators demonstrated positive trends, with 60% of patients perceiving improvements in service quality at PPP facilities, attributed to advanced technologies such as electronic health records, robotic surgery, and data-driven clinical protocols introduced under Vision 2030′s modernisation initiative [11,23]. These findings are consistent with global evidence that PPPs contribute to quality improvements through private-sector innovation and technological adoption. Brazil’s PPP clinics achieved a 15% reduction in chronic-disease complications through advanced diagnostics and telemedicine [24]; Portugal’s PPPs were associated with lower error rates (*p* < 0.01) and enhanced clinical responsiveness [12]; and integrated PPPs worldwide were associated with reduced wait times by 20% (*p* < 0.05) in resource-limited settings [33,34]. Italy’s Lab@AOR initiative delivered laboratory results 30% faster through automation [13], reinforcing the potential for Saudi Arabia to lead the region in healthcare technology adoption.

Operational challenges nevertheless temper these gains. In the present study, 52% of professionals reported increased patient volumes and sporadic delays, suggesting capacity strain—a phenomenon echoed in the United Kingdom, where PPPs improved infrastructure but faced staffing shortages and longer wait times due to cost-containment measures [7,35]. This reflects a broader critique that over-commercialisation risks prioritising profit over patient-centred care [34]. Scoping reviews of PPPs in primary health care highlight mixed and context-dependent effects on quality, with persistent challenges around governance, management, and health information systems. In low- and middle-income countries, health-system strengthening efforts often face fragmented programmes and uneven implementation standards, which can limit consistent quality gains [14]. A balanced-scorecard-type approach that integrates patient experience, clinical outcomes, and efficiency indicators into PPP performance assessment could help mitigate these risks, drawing on Kaplan and Norton’s balanced scorecard and the OECD’s renewed health-system performance-assessment framework [23,25]. Workforce capacity remains critical: training gaps reflect broader evidence that sustained quality improvement relies on structured continuing professional development (CPD) for health workers. Studies from Canada show that increased participation in accredited CPD correlates with higher-quality clinical practice [36], while work from India and other low- and middle-income settings highlights how uneven access to CPD and context-specific barriers hinder efforts to enhance the quality of care [37,38]. Mandating structured, competency-based CPD within PPP contractual frameworks, alongside the deliberate formation of interdisciplinary teams that integrate private-sector technologists with public-health practitioners, would help ensure that quality gains extend beyond capital investment in equipment to sustained, patient-centred models of care explicitly aligned with Vision 2030 priorities.

### 5.3. Financial and Operational Sustainability of PPPs

Sustainability findings in this study were cautiously favourable: 52.7% of professionals and 56.6% of patients perceived PPP-delivered healthcare as sustainable, and the regression analysis suggested that greater operational efficiency was associated with stronger fiscal performance. These findings should nevertheless be interpreted alongside the persistent financial barriers reported above, which indicate that sustainability gains have not yet translated into broader affordability for end-users. Evaluations of Lesotho’s national referral hospital PPP similarly reported improved technical efficiency and a lower cost per patient than under the previous public arrangements, but also highlighted substantial, ultimately unsustainable budgetary pressures [15,16]. International reviews of healthcare PPPs emphasise that efficiency gains and value for money are highly context-dependent and depend on contract design, risk allocation, and robust public-sector stewardship [39]. Evidence on greening health facilities shows that digital and infrastructure interventions can reduce energy use and operating costs when embedded in comprehensive energy-efficiency programmes [40,41]. Analyses of the COVID-19 period further underline that predictable funding, and resilient financing arrangements are critical for maintaining PPP service delivery during shocks [42,43].

Saudi Arabia’s dependence on volatile oil revenues makes ongoing large state subsidies for health-sector PPPs a potential fiscal risk, especially in the context of Vision 2030′s diversification agenda [19,44]. International evidence indicates that budget constraints are a key driver of PPP adoption and can limit their scalability as public finances tighten [10,45]. Resource Dependence Theory suggests that such vulnerability to a single revenue source can be reduced through interdependent partnerships that mobilise private capital, technology, and managerial capacity [45].

Examples such as Singapore show how combining mandatory savings, insurance schemes, and targeted subsidies can rebalance public–private roles while keeping government health expenditure relatively contained as a share of GDP [22]. For Saudi Arabia, options such as tiered co-payments for non-essential services, pooled public–private risk-sharing arrangements, and tax incentives for low-carbon health facilities could diversify revenue while protecting access for low-income groups, provided they are carefully designed and evaluated through pilot implementations [19,44].

### 5.4. Recommendations

Five strategic recommendations are derived directly from the empirical findings discussed above. Each recommendation is explicitly linked to the section that reports the underlying evidence, so the chain from finding to action remains transparent.

Recommendation 1—Regulatory enhancement (linked to Section 5.2 and Section 5.3): A comprehensive legal framework with standardised contract regulations and robust accountability mechanisms is needed to address the governance and regulatory ambiguities reported by professionals.

Recommendation 2—Targeted accessibility incentives (linked to Section 5.1): Targeted PPP incentives for rural healthcare expansion and telemedicine initiatives are required to close the urban–rural access gap (*p* = 0.008) and the persistent financial-affordability gap (mean = 2.2–2.3) documented in this study.

Recommendation 3—Performance-based contracts and CPD (linked to Section 5.2): Performance-based contracts focused on patient outcomes and competency-based continuing professional development should be embedded in PPP contractual frameworks to address the workforce-related quality concerns identified in the qualitative analysis.

Recommendation 4—Diversified financing and risk-sharing (linked to Section 5.3): Diversified financing strategies, including risk-sharing arrangements and pooled public–private cost models inspired by the Singapore experience, are necessary to mitigate concerns about subsidy dependence raised in the sustainability findings.

Recommendation 5—Community engagement and transparency (linked to Section 4.7): Greater transparency in PPP planning and execution, supported by public awareness campaigns, addresses the community engagement and information asymmetry concerns expressed in the qualitative thematic analysis.

### 5.5. Strengths and Limitations

This study has notable methodological strengths. A mixed-methods design enabled a comprehensive assessment of PPPs, and a stratified-pragmatic (quota-based) sampling design with explicit regional and facility-type quotas improved representation across stakeholder groups. Integrating primary and secondary data, applying expert-panel content validity assessment (CVI = 0.91), forward- and back-translation procedures, an exploratory factor-analytic check on the pilot data, and inter-coder reliability assessment for the qualitative strand (κ = 0.83) strengthened validity and reliability.

Several limitations should nevertheless be noted. First, the cross-sectional design precludes causal inference; the reported associations should be interpreted as correlational rather than causal. The perceived “before–after” comparisons depend on respondents’ retrospective recollection and are subject to recall bias. Second, although stratification quotas were enforced, recruitment relied on institutional gatekeepers and online channels, which may introduce selection bias; rural representation remains comparatively limited. Third, self-reported data are vulnerable to social desirability bias. Fourth, concurrent regulatory reforms within Vision 2030 may alter PPP performance beyond this study’s timeframe. Fifth, the regression models rely on observed predictors and cannot rule out residual confounding, although adjustment for age, sex, education, household income, comorbidities, and facility type partially mitigates this concern. Future longitudinal studies, comparative Gulf-region analyses, structural equation modelling and path analyses, and economic value-for-money evaluations are needed to inform evidence-based PPP policy aligned with Vision 2030.

## 6. Conclusions

This study provides empirical evidence that Public–Private Partnerships were associated with measurable perceived improvements in healthcare accessibility, quality, and operational efficiency in Saudi Arabia, supporting the aims of Vision 2030. The implementation of PPPs was associated with increased service reach, especially through telemedicine, and with better self-reported clinical experience driven by technological adoption. However, significant challenges remain, including unequal rural coverage, financial barriers to access worsened by out-of-pocket costs, capacity overload, and long-term fiscal dependency on government subsidies. The adjusted regression analysis identified financial accessibility and shorter waiting times as the variables most strongly associated with patient-reported outcomes and satisfaction, highlighting the importance of affordability and efficiency for PPP success.

For healthcare managers, the findings suggest the value of embedding competency-based continuing professional development into PPP contracts, deploying balanced-scorecard performance monitoring that integrates patient experience, clinical outcomes, and efficiency indicators, and strengthening interdisciplinary teamwork between private-sector technologists and public-sector clinicians.

For policymakers, the findings support: (i) the development of a comprehensive PPP legal framework with standardised contracts and transparent accountability mechanisms; (ii) tiered co-payment schemes and targeted financial-protection policies to mitigate persistent out-of-pocket burdens; (iii) rural-incentive mechanisms and broadband-expansion partnerships to close the urban–rural gap; (iv) pooled public–private risk-sharing instruments and tax incentives modelled on the Singapore experience to reduce subsidy dependence; and (v) workforce-nationalisation strategies that combine training pipelines with PPP-aligned employment incentives.

Future research should prioritise: (i) longitudinal designs that track PPP performance across multiple Vision 2030 milestones; (ii) comparative studies across the Gulf Cooperation Council to identify transferable lessons; (iii) advanced quantitative techniques such as structural equation modelling and path analysis to test mediating mechanisms among accessibility, quality, and sustainability constructs; and (iv) economic evaluations assessing value for money and equity trade-offs of alternative PPP modalities.

## Figures and Tables

**Table 1:** Reliability assessment by survey domain and cohort, including item-level statistics.

Domain	Item	Corr. Item–Total (r)	α If Deleted	M	SD	Subscale α
Accessibility	Ease of access after PPP implementation	0.62	0.74	3.20	1.10	0.78
	Telemedicine availability and usefulness	0.58	0.75	2.70	0.80	
	Out-of-pocket/financial accessibility	0.55	0.76	2.30	1.00	
	Distance and transport to facilities	0.51	0.77	2.60	1.10	
Quality	Patient outcomes and clinical effectiveness	0.68	0.78	3.10	1.00	0.82
	Waiting times	0.64	0.79	2.70	0.90	
	Safety and hygiene standards	0.61	0.80	3.90	0.80	
	Modern equipment and continuity of care	0.59	0.81	3.10	0.90	
Sustainability	Financial viability of PPP models	0.55	0.67	3.40	1.00	0.71
	Operational efficiency	0.52	0.68	2.70	0.90	
	Resilience and adaptability	0.49	0.69	2.60	1.00	
	Policy support and community engagement	0.46	0.70	2.80	1.00	

Note. Item-level statistics are presented for the healthcare-professional cohort. Patient-cohort subscale alphas were 0.76 (accessibility), 0.82 (quality), and 0.74 (sustainability).

**Table 2:** Inferential statistical analyses (n = 210).

Test	Comparison	Statistics	*p*-Value
*T*-test	Ease of access: <10 km vs. >30 km	2.8 vs. 2.1	0.008 **
*T*-test	Safety satisfaction: high vs. low frequency	2.9 vs. 2.6	0.038 *
ANOVA	Patient outcomes by frequency	F = 3.25	0.023 *
ANOVA	Financial viability by distance	F = 4.35	0.005 **
Chi-square	Safety satisfaction × distance	χ^2^ = 6.15	0.013 *
Chi-square	Telemedicine × frequency	χ^2^ = 7.02	0.008 **

Note. * *p* < 0.05; ** *p* < 0.01.

**Table 3:** Multiple regression analysis: variables associated with patient-reported outcomes (n = 210; fully adjusted model).

Predictor	B	SE	β	95% CI	t	*p*	VIF
Financial affordability	0.29	0.08	0.31	[0.13, 0.45]	3.65	<0.001 ***	1.42
Ease of access	0.25	0.08	0.27	[0.10, 0.40]	3.20	0.002 **	1.58
Reduced waiting times	0.21	0.07	0.23	[0.06, 0.36]	2.85	0.005 **	1.31
Distance from facility	−0.14	0.06	−0.16	[−0.26, −0.02]	−2.28	0.024 *	1.27
Age	0.02	0.04	0.04	[−0.06, 0.10]	0.55	0.581	1.18
Sex (female)	0.06	0.07	0.05	[−0.08, 0.20]	0.85	0.396	1.08
Education	0.08	0.05	0.10	[−0.02, 0.18]	1.62	0.107	1.46
Household income	0.10	0.05	0.12	[0.00, 0.20]	1.95	0.052	1.51
Comorbidity count	−0.05	0.05	−0.06	[−0.15, 0.05]	−0.95	0.343	1.22
Facility type (private)	0.07	0.07	0.06	[−0.07, 0.21]	1.02	0.309	2.31

Note. R^2^ = 0.41, Adjusted R^2^ = 0.39, F = 28.6, *p* < 0.001. * *p* < 0.05; ** *p* < 0.01; *** *p* < 0.001. B = unstandardised regression coefficient (change in the outcome score on the 1–5 Likert scale per one-unit change in the predictor, holding other variables constant); SE = standard error of B; β = standardised regression coefficient; 95% CI = 95% confidence interval for B; t = t-statistic for B; *p* = *p*-value; VIF = Variance Inflation Factor. R^2^ = 0.41, Adjusted R^2^ = 0.39, F = 28.6, *p* < 0.001. * *p* < 0.05; ** *p* < 0.01; *** *p* < 0.001. Predictors selected based on theoretical relevance and bivariate screening (*p* < 0.10). VIF < 5 indicates the absence of problematic multicollinearity.

**Table 4:** Stratified regression coefficients (β) by patient subsample.

Predictor	Urban (n = 128)	Rural (n = 82)	Government (n = 137)	Private (n = 73)
Financial affordability	0.27 **	0.38 ***	0.29 **	0.36 ***
Ease of access	0.30 **	0.21 *	0.28 **	0.24 *
Reduced waiting times	0.28 **	0.15	0.26 **	0.18 *
Distance from facility	−0.10	−0.22 *	−0.14 *	−0.19 *

Note. * *p* < 0.05; ** *p* < 0.01; *** *p* < 0.001. Each model adjusts for age, sex, education, household income, comorbidity count, and (for facility-type strata) urban/rural location.

## Data Availability

Data available upon request due to ethical restrictions.

## Supplementary Materials

The following supporting information can be downloaded at: https://www.mdpi.com/article/10.3390/healthcare14111435/s1, A completed SRQR checklist, A completed COREQ checklist, A reflexivity statement.

## Abbreviations

The following abbreviations are used in this manuscript:

PPP	Public–Private Partnership
ANOVA	Analysis of Variance
OECD’s	Organization for Economic Co-operation and Development
CPD	Continuing Professional Development
GPD	Gross Domestic Product

## Appendix A. Survey Items

Appendix A presents the full questionnaire, organised by section, with item code, item wording, brief description, and measurement scale. Likert items are scored from 1 (very dissatisfied/strongly disagree/much worse) to 5 (very satisfied/strongly agree/much better), unless otherwise noted.

**Section**	**Code**	**Item**	**Description**	**Scale**
Demographics	D1	Role in the healthcare system	Manager/physician/nurse/technician/pharmacist/policymaker patient	Categorical
Demographics	D2	Years of professional experience (or age, for patients)	Continuous in years; ordinal bands of 5 years used for analysis	Ordinal
Demographics	D3	Employment sector	Government/private	Categorical
Demographics	D4	Region of residence/work	Central/Western/Eastern/ Northern/Southern	Categorical
Demographics	D5	Frequency of healthcare utilisation (patients only)	Rarely/sometimes/often/ very frequently	Ordinal
Accessibility	A1	Ease of access after PPP implementation	Self-rated change in physical access	Likert 1–5
Accessibility	A2	Availability of specialised services	Perceived breadth of specialist availability	Likert 1–5
Accessibility	A3	Availability of transport/distance to facility	Perceived ease of reaching the facility	Likert 1–5
Accessibility	A4	Financial accessibility/out-of-pocket costs	Perceived change in affordability	Likert 1–5
Accessibility	A5	Patient navigation support	Availability of guidance services	Likert 1–5
Accessibility	A6	Telemedicine and digital-health solutions	Use and usefulness of digital channels	Likert 1–5
Accessibility	A7	Distance band to nearest PPP facility	<10 km/10–30 km/>30 km	Categorical
Quality	Q1	Patient outcomes and clinical effectiveness	Perceived change in outcomes	Likert 1–5
Quality	Q2	Availability of modern equipment	Perceived modernisation level	Likert 1–5
Quality	Q3	Waiting times	Perceived change in waiting times	Likert 1–5
Quality	Q4	Safety and hygiene standards	Adherence to infection-control standards	Likert 1–5
Quality	Q5	Staff training and competence	Perceived staff preparedness	Likert 1–5
Quality	Q6	Continuity of care	Coordination across services	Likert 1–5
Quality	Q7	Service quality (overall)	Overall perceived quality of care	Likert 1–5
Quality	Q8	Effectiveness of care under PPPs	Perceived effectiveness of treatment	Likert 1–5
Sustainability	S1	Financial viability of PPP models	Perceived financial sustainability	Likert 1–5
Sustainability	S2	Operational efficiency	Perceived efficiency of operations	Likert 1–5
Sustainability	S3	Environmental responsibility	Energy and waste-management practices	Likert 1–5
Sustainability	S4	Social responsibility/community focus	Engagement with local communities	Likert 1–5
Sustainability	S5	Resilience and adaptability	Capacity to respond to shocks	Likert 1–5
Sustainability	S6	Policy and regulatory support	Adequacy of regulatory framework	Likert 1–5
Sustainability	S7	Community engagement	Participation of users in governance	Likert 1–5
Sustainability	S8	Alignment with Vision 2030	Perceived alignment with national strategy	Likert 1–5
Open feedback	O1	Main barriers experienced under PPPs	Free-text response	Open-ended
Open feedback	O2	Suggestions for improvement	Free-text response	Open-ended
Open feedback	O3	Perceived alignment with Vision 2030	Not aligned/somewhat aligned/ very aligned	Categorical
Open feedback	O4	Most valuable PPP feature experienced	Free-text response	Open-ended
Open feedback	O5	Most pressing concern about PPPs	Free-text response	Open-ended
