# Supplementary material for: Public–Private Partnerships as a Catalyst for Healthcare Transformation in Saudi Arabia: Evaluating the Impact on Accessibility, Quality, and Sustainability Under Vision 2030
Source: Healthcare (Basel). 2026 May 22;14(11):1435. doi: 10.3390/healthcare14111435 (PMC13257269; doi:10.3390/healthcare14111435)
Supplement: Supplementary file 1 [file healthcare-14-01435-s001.zip › healthcare-4228721-supplementary.pdf]

Supplementary Material 1

SRQR — Standards for Reporting Qualitative Research

Completed checklist for the manuscript: Public–Private Partnerships as a Catalyst for Healthcare Transformation in Saudi Arabia

Reference: O'Brien BC, Harris IB, Beckman TJ, Reed DA, Cook DA. Standards for reporting qualitative research: a synthesis of recommendations. Acad Med. 2014;89(9):1245–51. doi:10.1097/ACM.0000000000000388.

The following table reports the SRQR's 21 items and identifies, for each item, where in the manuscript and supplementary materials the corresponding information can be found.

No.	SRQR item / topic	Item description	Reported in
**Title and abstract**			
1	**Title**	*Concise description of the nature and topic of the study identifying the study as qualitative or indicating the approach (e.g., ethnography, grounded theory) or data-collection methods (e.g., interview, focus group) is recommended.*	Title page. The full title indicates a mixed-methods evaluation; the qualitative component is identified in the Methods (Section 3.1) and the Abstract.
2	**Abstract**	*Summary of key elements of the study using the abstract format of the intended publication; typically includes background, purpose, methods, results, and conclusions.*	Abstract (page 1). The qualitative thematic analysis, the inter-coder reliability statistic (κ = 0.83), and the open-ended response procedure are summarised.
**Introduction**			
3	**Problem formulation**	*Description and significance of the problem/phenomenon studied; review of relevant theory and empirical work; problem statement.*	Section 1 (Introduction) and Section 2 (Literature Review and Theoretical Framework). The theoretical gap regarding healthcare PPPs in oil-dependent emerging economies is articulated.
4	**Purpose or research question**	*Purpose of the study and specific objectives or questions.*	Section 1 (final paragraph). The explicit research question, four hypotheses (H1–H4), and the dedicated purpose statement are presented.
**Methods**			
5	**Qualitative approach and research paradigm**	*Qualitative approach (e.g., ethnography, grounded theory, case study, phenomenology, narrative research) and guiding theory if appropriate; identifying the research paradigm (e.g., postpositivist,*	Section 3.5 (Data Analysis — Qualitative Analysis). The study uses a thematic-analysis approach following Braun and Clarke (2006), within an interpretivist paradigm consistent with the integrated

No.	SRQR item / topic	Item description	Reported in
		constructivist/interpretivist) is also recommended.	theoretical framework presented in Section 2 (Health Equity Model + Resource Dependence Theory + OECD HSPA).
6	**Researcher characteristics and reflexivity**	*Researchers' characteristics that may influence the research, including personal attributes, qualifications/experience, relationship with participants, assumptions, and/or presuppositions; potential or actual interaction between researchers' characteristics and the research questions, approach, methods, results, and/or transferability.*	Section 3.5 (Qualitative Analysis) and Reflexivity Statement (Supplementary Material). Both researchers are health-services academics with prior experience in PPP and Saudi healthcare research. Their disciplinary lens is acknowledged; no clinical relationships with participants existed.
7	**Context**	*Setting/site and salient contextual factors; rationale.*	Section 3.1 (Study Design) and Section 3.2 (Participants, Sampling, and Recruitment). PPP-operated facilities across the five administrative regions of Saudi Arabia are described, situated within the Vision 2030 reform context.
8	**Sampling strategy**	*How and why research participants, documents, or events were selected; criteria for deciding when no further sampling was necessary (e.g., sampling saturation); rationale.*	Section 3.2. Stratified-pragmatic (quota-based) sampling with convenience elements; quotas across regions and facility types; sampling continued until quota targets were met. Rationale: balance representativeness with feasibility in an emerging-economy survey context.
9	**Ethical issues pertaining to human subjects**	*Documentation of approval by an appropriate ethics review board and participant consent, or explanation for lack thereof; other confidentiality and data security issues.*	Section 3.7 (Ethical Considerations). IRB approval from King Fahad Medical City (Log Number 25-7001, 16 February 2026). Informed electronic consent obtained; data anonymised and stored on a secure institutional server in compliance with the Saudi Personal Data Protection Law (PDPL).
10	**Data collection methods**	*Types of data collected; details of data collection procedures including (as appropriate) start and stop dates of data collection and analysis, iterative process, triangulation of sources/methods, and modification of procedures in response to evolving study findings; rationale.*	Section 3.4 (Survey Content) and Section 3.5. Open-ended questions were embedded in the structured online survey. Data collection ran from February to April 2026. Triangulation between quantitative and qualitative strands is reported via

No.	SRQR item / topic	Item description	Reported in
			Spearman rank correlation (ρ = 0.80) in Section 4.7.
11	**Data collection instruments and technologies**	*Description of instruments (e.g., interview guides, questionnaires) and devices (e.g., audio recorders) used for data collection; if/how the instrument(s) changed over the course of the study.*	Section 3.4 and Appendix A. The survey instrument (38 items) included five open-ended questions. Items were developed from validated PPP and health-services instruments, expert-reviewed (CVI = 0.91), forward- and back-translated (English/Arabic), and pilot-tested (n = 28). Two items were refined after pilot testing.
12	**Units of study**	*Number and relevant characteristics of participants, documents, or events included in the study; level of participation (could be reported in results).*	Section 4.1 (Participant Demographics). 360 valid responses analysed: 150 healthcare professionals (60.7% practitioners, 8.7% policymakers, 8.7% government officials, 6.0% researchers, 4.0% hospital managers, 2.0% nurses, 0.7% technicians) and 210 patients.
13	**Data processing**	*Methods for processing data prior to and during analysis, including transcription, data entry, data management and security, verification of data integrity, data coding, and anonymization/de-identification of excerpts.*	Section 3.5 (Qualitative Analysis). Open-ended responses were exported from the survey platform, anonymised at source (no identifiers were collected), imported into NVivo 14, and coded inductively. Data integrity was verified through random spot-checks.
14	**Data analysis**	*Process by which inferences, themes, etc., were identified and developed, including the researchers involved in data analysis; usually references a specific paradigm or approach; rationale.*	Section 3.5. Six-phase Braun and Clarke (2006) thematic-analysis procedure: familiarisation, initial coding, theme generation, theme review, theme definition, and reporting. Two researchers coded independently; discrepancies resolved by discussion with a third researcher when necessary.
15	**Techniques to enhance trustworthiness**	*Techniques to enhance trustworthiness and credibility of data analysis (e.g., member checking, audit trail, triangulation); rationale.*	Section 3.5 and Section 4.7. Trustworthiness was supported by: (i) inter-coder reliability assessment (Cohen's κ = 0.83, 95% CI 0.78–0.87); (ii) audit trail maintained in NVivo 14; (iii) methodological triangulation via Spearman correlation between qualitative theme frequencies and

No.	SRQR item / topic	Item description	Reported in
			quantitative dimension means (ρ = 0.80); (iv) reflexivity statement.
**Results / Findings**			
16	**Synthesis and interpretation**	*Main findings (e.g., interpretations, inferences, and themes); might include development of a theory or model, or integration with prior research or theory.*	Section 4.7 (Qualitative Thematic Analysis). Six themes identified: financial concerns; access and equity; quality and services; policy and regulatory; workforce and training; community engagement. Findings integrated with the theoretical framework in Section 5.
17	**Links to empirical data**	*Evidence (e.g., quotes, field notes, text excerpts, photographs) to substantiate analytic findings.*	Section 4.7. Six representative anonymised excerpts illustrate each theme (one per theme). Theme frequencies reported in Figure 7.
**Discussion**			
18	**Integration with prior work, implications, transferability, and contribution(s) to the field**	*Short summary of main findings; explanation of how findings and conclusions connect to, support, elaborate on, or challenge conclusions of earlier scholarship; discussion of scope of application/generalizability; identification of unique contribution(s) to scholarship in a discipline or field.*	Sections 5.1–5.4. Findings are situated within international PPP literature (UK PFI, Lesotho, Brazil, India, Turkey, Portugal, Italy, Singapore). The study contributes the first integrated equity-RDT-OECD framework to a Gulf-region empirical PPP evaluation. Transferability is discussed for oil-dependent emerging economies.
19	**Limitations**	*Trustworthiness and limitations of findings.*	Section 5.5 (Strengths and Limitations). Cross-sectional design precludes causal inference; recall and social-desirability bias possible; rural representation comparatively limited; concurrent regulatory reforms; reliance on observed predictors. The qualitative component is limited to short open-ended responses rather than full interviews.
**Other**			
20	**Conflicts of interest**	*Potential sources of influence or perceived influence on study conduct and conclusions; how these were managed.*	Conflicts of Interest statement (back matter). The authors declare no conflicts of interest.

No.	SRQR item / topic	Item description	Reported in
**21**	**Funding**	*Sources of funding and other support; role of funders in data collection, interpretation, and reporting.*	Funding statement (back matter). This research received no external funding.

Supplementary Material 2

COREQ — Consolidated Criteria for Reporting Qualitative Research

Completed 32-item checklist for the manuscript: Public–Private Partnerships as a Catalyst for Healthcare Transformation in Saudi Arabia

Reference: Tong A, Sainsbury P, Craig J. Consolidated criteria for reporting qualitative research (COREQ): a 32-item checklist for interviews and focus groups. Int J Qual Health Care. 2007;19(6):349–57. doi:10.1093/intqhc/mzm042.

Note on applicability: The qualitative component of this study consisted of open-ended responses embedded within a structured online survey rather than face-to-face interviews or focus groups. The COREQ checklist is therefore reported here in adapted form: items pertaining to interviewer presence, audio recording, and transcript return are marked as not applicable, and the corresponding methodological choices and limitations are reported transparently below and in Section 5.5 of the manuscript.

No.	COREQ item / topic	Guide question / description	Reported in / Response
**Domain 1: Research team and reflexivity**			
**Personal characteristics**			
**1**	**Interviewer/facilitator**	*Which author/s conducted the interview or focus group?*	Open-ended responses were collected via the structured online survey rather than face-to-face interviews; therefore no interviewer was directly present during data collection. Both authors (S.B. and M.A.) coded the responses (Section 3.5).
**2**	**Credentials**	*What were the researcher's credentials? E.g., PhD, MD*	Both researchers hold doctoral-level qualifications in healthcare-related disciplines (radiology and medical imaging; interventional radiology). Acknowledgements section.
**3**	**Occupation**	*What was their occupation at the time of the study?*	Academic/clinical researchers affiliated with King Fahad Medical City and Prince Sattam Bin Abdulaziz University. Title page.
**4**	**Gender**	*Was the researcher male or female?*	Both researchers are male. Reflexivity Statement (Supplementary Material).
**5**	**Experience and training**	*What experience or training did the researcher have?*	Both researchers have prior experience in health-services research and qualitative coding. Training in NVivo 14 and the Braun and Clarke (2006) thematic-analysis procedure was

No.	COREQ item / topic	Guide question / description	Reported in / Response
			undertaken before the study. Reflexivity Statement.
**Relationship with participants**			
6	**Relationship established**	*Was a relationship established prior to study commencement?*	No personal relationship existed between the researchers and individual participants. Section 3.2 and Reflexivity Statement.
7	**Participant knowledge of the interviewer**	*What did the participants know about the researcher? E.g., personal goals, reasons for doing the research*	The introductory page of the survey disclosed the researchers' affiliations, the academic purpose of the study, and its alignment with Vision 2030 healthcare-evaluation objectives. Section 3.2.
8	**Interviewer characteristics**	*What characteristics were reported about the interviewer/facilitator? E.g., bias, assumptions, reasons and interests in the research topic*	The researchers' disciplinary backgrounds, prior experience with Saudi healthcare reform, and methodological assumptions are disclosed in the Reflexivity Statement (Supplementary Material).
**Domain 2: Study design**			
**Theoretical framework**			
9	**Methodological orientation and Theory**	*What methodological orientation was stated to underpin the study?*	Reflexive thematic analysis (Braun and Clarke, 2006), within an interpretivist paradigm and the integrated theoretical framework presented in Section 2 (Health Equity Model + Resource Dependence Theory + OECD HSPA). Section 3.5.
**Participant selection**			
10	**Sampling**	*How were participants selected? E.g., purposive, convenience, consecutive, snowball*	Stratified-pragmatic (quota-based) sampling with convenience elements; quotas applied by region and facility type. Section 3.2.
11	**Method of approach**	*How were participants approached? E.g., face-to-face, telephone, mail, email*	Email and institutional gatekeepers (hospital and clinic administrators); time-staggered survey dissemination. Section 3.2.
12	**Sample size**	*How many participants were in the study?*	150 healthcare professionals and 210 patients (n = 360 valid responses out of 565

No.	COREQ item / topic	Guide question / description	Reported in / Response
			invitations). Section 3.2 and Figure 3 (STROBE flow).
13	**Non-participation**	*How many people refused to participate or dropped out? Reasons?*	205 invitees did not respond or were excluded (95 professionals: 78 non-response, 9 duplicates, 8 incomplete; 110 patients: 92 non-response, 11 duplicates, 7 incomplete). Figure 3.
**Setting**			
14	**Setting of data collection**	*Where was the data collected? E.g., home, clinic, workplace*	Online (self-administered) survey accessible to participants in any location with internet access. Section 3.2.
15	**Presence of non-participants**	*Was anyone else present besides the participants and researchers?*	Not applicable. Self-administered online responses; no facilitator or third party was present during completion. Section 3.2.
16	**Description of sample**	*What are the important characteristics of the sample? E.g., demographic data, date*	Professionals: 60.7% practitioners, 8.7% policymakers, 8.7% government officials, 6.0% researchers, 4.0% managers, 2.0% nurses, 0.7% technicians; 65.3% government sector. Patients: 60% frequent users; geographically distributed across five administrative regions. Section 4.1; Figure 2.
**Data collection**			
17	**Interview guide**	*Were questions, prompts, guides provided by the authors? Was it pilot tested?*	Yes. The five open-ended questions are listed in Appendix A (items O1–O5). The instrument was pilot-tested with 28 respondents; two ambiguous items were refined. Sections 3.4 and 3.5.
18	**Repeat interviews**	*Were repeat interviews carried out? If yes, how many?*	Not applicable. Single cross-sectional survey. Section 3.1.
19	**Audio/visual recording**	*Did the research use audio or visual recording to collect the data?*	Not applicable. Open-ended responses were typed directly into the survey platform. Section 3.5.
20	**Field notes**	*Were field notes made during and/or after the interview or focus group?*	Not applicable. Methodological notes were maintained in the NVivo 14 audit trail during coding. Section 3.5.

No.	COREQ item / topic	Guide question / description	Reported in / Response
21	**Duration**	*What was the duration of the interviews or focus group?*	Median completion time of the full survey was approximately 15 minutes (range 9–28 minutes), based on platform-recorded timestamps. Section 3.2.
22	**Data saturation**	*Was data saturation discussed?*	Theme saturation was monitored across coding cycles. After approximately 75% of the open-ended responses had been coded, no new themes emerged; remaining responses were used to refine and confirm theme boundaries. Section 3.5.
23	**Transcripts returned**	*Were transcripts returned to participants for comment and/or correction?*	Not applicable. Responses were typed directly by participants; no transcription stage was needed. Member checking was not performed because of anonymous data collection. Limitation noted in Section 5.5.
**Domain 3: Analysis and findings**			
**Data analysis**			
24	**Number of data coders**	*How many data coders coded the data?*	Two researchers coded all open-ended responses independently. Section 3.5.
25	**Description of the coding tree**	*Did authors provide a description of the coding tree?*	Six top-level themes are reported in Section 4.7 with sub-codes summarised in the qualitative thematic frequency analysis (Figure 7). The full coding tree is available from the corresponding author on request.
26	**Derivation of themes**	*Were themes identified in advance or derived from the data?*	Themes were derived inductively from the data, then mapped onto the integrated theoretical framework (Section 2) for interpretive synthesis. Section 3.5.
27	**Software**	*What software, if applicable, was used to manage the data?*	NVivo 14 was used for qualitative thematic coding. IBM SPSS Statistics version 29 was used for quantitative analyses; R 4.4 (ggplot2) for visualisations. Section 3.1.
28	**Participant checking**	*Did participants provide feedback on the findings?*	Not performed; the survey was anonymous and participant

No.	COREQ item / topic	Guide question / description	Reported in / Response
			identities were not retained. Limitation noted in Section 5.5.
**Reporting**			
**29**	**Quotations presented**	*Were participant quotations presented to illustrate the themes/findings? Was each quotation identified? E.g., participant number*	Yes. Six representative anonymised excerpts (one per theme) are reported in Section 4.7. Each excerpt is identified by participant role (e.g., "a patient observed", "a hospital manager noted") to preserve anonymity.
**30**	**Data and findings consistent**	*Was there consistency between the data presented and the findings?*	Yes. Convergence between qualitative themes and quantitative dimensions is documented through the Spearman rank correlation (ρ = 0.80) reported in Section 4.7 and discussed in Section 5.
**31**	**Clarity of major themes**	*Were major themes clearly presented in the findings?*	Yes. The six major themes (financial concerns; access and equity; quality and services; policy and regulatory; workforce and training; community engagement) are presented in Section 4.7 and visualised in Figure 7.
**32**	**Clarity of minor themes**	*Is there a description of diverse cases or discussion of minor themes?*	Yes. Differences between professional and patient perspectives within each theme (e.g., systemic vs immediate financial concerns; differing emphasis on community engagement) are reported in Section 4.7 and discussed in Section 5.

Supplementary Material 3
Reflexivity Statement

Researcher Positionality

Both authors are male academic and clinical researchers based in Saudi Arabia, with doctoral qualifications in radiology and medical imaging. We have prior experience in health services research and professional familiarity with both public-sector and PPP-operated healthcare facilities under Vision 2030. We conducted the qualitative analysis within an interpretivist paradigm. We were guided by the integrated theoretical framework presented in Section 2 of the manuscript (Health Equity Model + Resource Dependence Theory + OECD Health System Performance Assessment).

Relationship with Participants

No prior personal relationship existed between the researchers and individual participants. Recruitment was conducted through institutional gatekeepers (hospital and clinic administrators) and online channels. All data collection was anonymous; participants were aware of the study's academic purpose and its alignment with Vision 2030 healthcare-evaluation objectives, but were not aware of the researchers' specific theoretical commitments.

Pre-existing Assumptions

Before data collection, we held three working assumptions, drawn from the international literature on healthcare PPPs: (i) that PPPs would be associated with perceived improvements in physical access and technological adoption; (ii) that financial barriers would persist or worsen for some user groups; and (iii) that workforce and sustainability concerns would be more salient to professionals than to patients. These assumptions informed the sampling strategy and the variable-selection logic, but we deliberately did not predefine themes for the qualitative analysis. Themes were derived inductively from the open-ended responses (Section 3.5) and were then mapped onto the theoretical framework for interpretive synthesis.

Influence on Data Analysis

Both researchers independently coded the open-ended responses, and inter-coder reliability was assessed (Cohen's $\kappa = 0.83$, 95% CI 0.78–0.87). Discrepancies were resolved through structured discussion; when consensus could not be reached, a third researcher was consulted. To mitigate the risk that our shared institutional and disciplinary background might homogenise interpretation, we deliberately surfaced and discussed any patterns of agreement that emerged too quickly. We also maintained an audit trail in NVivo 14 documenting code definitions, theme-development decisions, and dissenting interpretations.

Acknowledged Limitations

We acknowledge that our positionality as Saudi-based health-services researchers may have influenced the salience we attached to certain themes (e.g., workforce nationalisation; subsidy dependence). Conversely, themes more salient to non-Saudi stakeholders or to vulnerable rural populations may have been under-represented. Member checking was not conducted because the survey was anonymous, and this is acknowledged as a limitation in Section 5.5 of the manuscript.
